# Effect of mastication evaluation and intervention on body composition and biochemical indices in female patients with obesity: a randomized controlled trial

**DOI:** 10.1186/s12902-023-01379-2

**Published:** 2023-06-21

**Authors:** Nagisa Hidaka, Satoshi Kurose, Nana Takao, Takumi Miyauchi, Sachiko Nakajima, Sawako Yoshiuchi, Aya Fujii, Kazuhisa Takahashi, Hiromi Tsutsumi, Daiki Habu, Kazuhiro Taniguchi, Yutaka Kimura

**Affiliations:** 1 Department of Health Science, Kansai Medical University, Hirakata, Osaka 573-1010 Japan; 2 Faculty of International Studies, Osaka Sangyo University, Daito, Osaka 574-8530 Japan; 3 Health Science Center, Kansai Medical University Hospital, Hirakata, Osaka 573-1010 Japan; 4 Department of Medicine II, Kansai Medical University, Hirakata, Osaka 573-1010 Japan; 5 Graduate School of Human Life and Ecology Division of Human Life and Ecology, Osaka Metropolitan University, Osaka-shi, Osaka 558-8585 Japan; 6 Faculty of Human Ecology, Department of Aesthetic Design and Technology, Yasuda Women’s University, Hiroshima-shi, Hiroshima 731-0153 Japan

**Keywords:** Mastication, Biochemical indices, Obesity, Nutritional guidance

## Abstract

**Background:**

A limited number of studies have evaluated the masticatory indices of individuals with obesity who only chew their food a few times and for shorter duration or who were provided with an instructional intervention. This study aimed to examine the effects of a 6-month instructional mastication intervention on the body composition and biochemical indices in female patients with obesity.

**Methods:**

Female patients with obesity were randomly classified into a conventional treatment group (CTG; 12 individuals), which only received normal nutritional and exercise guidance, and a mastication intervention group (MIG; 16 individuals), which received an additional mastication guidance. The MIG received guidance on foods requiring increased number of chews and chewing duration, eating techniques, and the proper method of cutting foods.

**Results:**

Changes in the masticatory, body composition, and biochemical indices were compared before and after the 6-month intervention. The values of body composition indices decreased significantly in both groups; however, the rate of change in body mass index significantly decreased in the MIG. In addition, the values of biochemical indices were significantly decreased in the MIG compared with that in the CTG, which is attributed to the addition of mastication instruction to female patients with obesity.

**Conclusion:**

Increasing the number of chews and duration of chewing times for carbohydrates, which are staple foods, possibly contributed to weight loss and improvement of glucose metabolism.

**Trial registration:**

UMIN, UMIN000025875. Registered on 27 Jan 2017.

## Background

Obesity is a well-established risk factor for many lifestyle-related conditions, including hypertension, diabetes, dyslipidemia, and cardiovascular disease [1]. Prevention and treatment of obesity are important for extending a healthy lifespan. Poor nutritional habits are one of the causes of obesity [2]. Exercise and nutritional interventions have been used for the prevention and treatment of obesity. Christen et al. hypothesized that adequate mastication could prevent binge eating [3]. However, the nutritional guidance provided in clinical practice only focused on adjustment of energy intake and maintenance of nutritional balance; hence, it remains unclear whether masticatory guidance has been adequately provided in the long term.

Recently, the importance of mastication has received increasing research attention. Eating speed, number of chews, and other eating behaviors and habits are also associated with obesity. Traditionally and supported by numerous studies, “fast eating,” with fewer chews and shorter meal duration, is one of the factors contributing to obesity. For example, college students who eat quickly are 4.4 times more likely to become obese, and men have a 2.8-fold higher risk of developing obesity than women [4]. Furthermore, the number of chews and chewing duration are closely related to energy expenditure, satiety [5, 6], and increased risk of developing obesity [7–9]; this finding suggests that nutritional guidance and methods for improving the number of chews and chewing duration may be useful.

Li et al. compared the masticatory behavior of lean male patients with obesity. The obese group exhibited a significantly lower number of chews per gram of food and a higher rate of ingestion and energy intake per gram of food [10]. Regardless of whether the participants were lean or obese, their energy intake was 11.9% lower when they chewed 40 times per meal than that when they chewed 15 times per meal, indicating that the interventions aimed at improving mastication can be useful in combating obesity. However, this was an experimental study involving patients with obesity and only lasted for 3 days. Therefore, the long-term effect of mastication instructional intervention on people with obesity needs to be evaluated further.

We have previously conducted a cross-sectional study to examine the relationship between mastication characteristics and obesity indices in patients with obesity. This relationship was established by comparing the number of chews and chewing duration for each food item in the obese and non-obese groups using a device with optical sensors plugged into the external auditory canal for measurement [11]. Patients with obesity demonstrated a significantly shorter chewing duration and lower chewing frequency. Sex-related differences in masticatory behavior were also revealed, suggesting that masticatory behavior even affects metabolism, particularly in women with obesity. In our cross-sectional study, the obese participants showed gender differences in mastication behavior; in particular, the number and duration of mastication in obese middle-aged and older women were associated with the glucose metabolism indices. Therefore, mastication guidance for obese middle-aged and elderly women can significantly improve the metabolic indices compared with the regular nutritional guidance.

In this study, we aimed to conduct a 6-month instructional mastication intervention in female patients with obesity and examine its effects on their body composition and biochemical indices.

## Methods

### Participants

Forty female obese patients with a body mass index (BMI) of ≥ 30 kg/m^2^ who visited an obesity outpatient clinic were enrolled in the study. After medical examination by a physician, the patients provided written informed consent to participate in this study. The preliminary measurements of body composition, biochemical indices, and masticatory indices were obtained. Subsequently, the patients were randomly classified into the conventional treatment group (CTG), which only received normal nutritional and exercise guidance, and the mastication intervention group (MIG), which received masticatory guidance in addition to nutritional and exercise guidance. The patients were randomly assigned to groups using envelopes. Medical personnel, who were not involved in the study designing, were assigned by drawing lots. Using G power, the number of cases was calculated by setting an effect size of 0.5, an α-error of 0.05, and a power of 0.80. Six patients with chewing disorders, with oral diseases, and under medication treatment were excluded. Of the 34 enrolled patients, three dropouts were identified from each group. Therefore, only 28 patients (12 and 16 in the CTG and MIG groups, respectively [age: 42.9 ± 12.5 years, BMI: 37.1 ± 6.0 kg/m^2^) (CTG: age = 40.5 ± 12 years, BMI = 37.4 ± 5.2 kg/m^2^; MIG: age = 44.6 ± 12.4 years, BMI = 36.9 ± 6.7 kg/m^2^) were included in the final analysis.

Patients aged ≥ 20 years and women who were able to perform their daily living activities independently with a BMI of ≥ 30 kg/m^2^ were included. Moreover, an understanding of the description of this study and provision of voluntary written consent were required. Individuals with masticatory problems due to a cranial nerve disorder and those with oral disease, otitis externa, or psychiatric disorders were deemed unsuitable for participation in the study by the principal investigator and were excluded. Participants who were diagnosed with diabetes only, but did not receive medication treatment were considered as having diabetes; meanwhile, those with non-alcoholic fatty liver were also considered as having fatty liver. Smoking, drinking, and exercise habits were defined according to the standards of the “National Health and Nutrition Survey” of the Ministry of Health, Labour and Welfare of Japan.

This study was approved by the Ethics Committee of Kansai Medical University (approval no. 1647) and the Ethics Committee of Osaka Sangyo University (application no. 2016-*Jinrin*-019).

### Study protocol

**Fig. 1:**
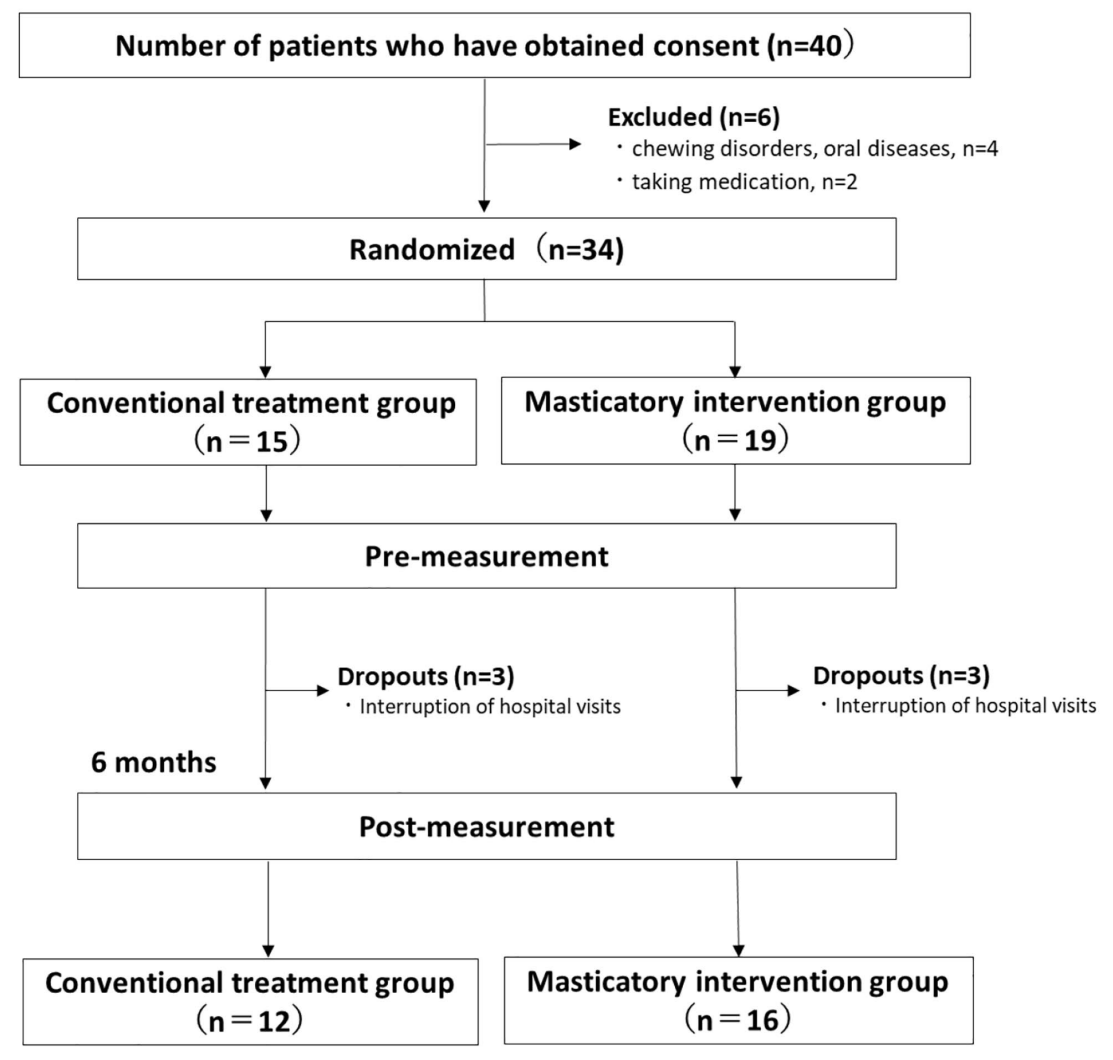
Research protocol

The body composition, biochemical, and masticatory indices of all patients were measured before and after the intervention (Fig. 1). Our Obesity Outpatient Department manages obese patients with a BMI of ≥ 30 kg/m^2^. After consultation with a physician, patients underwent a comprehensive 6-month weight loss program supervised by a registered dietitian, health and exercise instructor, and licensed psychologist at our outpatient department. Nutritional guidance by a registered dietitian and cognitive behavioral therapy by a psychologist were performed once a month for 6 months. In-hospital monitored exercise therapy was performed at least once a month. The exercise intervention consisted of 30 min of aerobic exercise at an intensity corresponding to the anaerobic threshold determined by cardiopulmonary exercise tests, three types of strength training exercises, and stretching. Furthermore, the goal was to perform exercise at home three or more times a week at the same intensity as that of the monitored exercise therapy. In both groups, cognitive behavioral therapy was provided, and patients independently set their own behavioral goals; the patients were guided on the appropriate nutritional balance and calorie intake. The MIG focused on the assessment of their masticatory behavior, in addition to the conventional treatment guidance; this group was specifically instructed on how to select foods that require more chews and longer chewing duration, how to prepare food, how to put down chopsticks after each bite, and how to chew > 30 times per bite. Furthermore, the degree of implementation of actionable mastication goals was assessed once a month during the provision of nutritional guidance. If implementation was difficult, the goals were re-set, and the participants were instructed to improve mastication.

### Body composition

Body composition was measured in a fasting state using InBody 720 (Biospace, Korea). The indices measured were body weight, body fat, and BMI (calculated by dividing weight in kilograms (kg) by height in meter squared (m^2^)). Furthermore, the visceral and subcutaneous fat areas were measured using computed tomography (CT; GE Healthcare, USA) to determine the cross-sectional area above the navel. CT and fat scan analysis software (East Japan Technology Tokyo Institute, Tokyo, Japan) were used to measure the visceral and subcutaneous fat areas at the navel level.

### Biochemical tests

Blood samples were collected early in the morning after an overnight fast. The biochemical indices used in this study were the levels of aspartate aminotransferase (AST), alanine aminotransferase (ALT), gamma-glutamyl transferase (GGT), fasting plasma glucose (FPG), hemoglobin A1c (HbA1c), triglyceride (TG), low-density lipoprotein (LDL), high-density lipoprotein (HDL), immunoreactive insulin (IRI), and homeostasis model assessment of insulin resistance (HOMA-R). HOMA-R was used to measure insulin resistance and was calculated using the following formula: resistance index = fasting insulin×fasting blood glucose/405.

### Assessment of mastication and food items

**Fig. 2:**
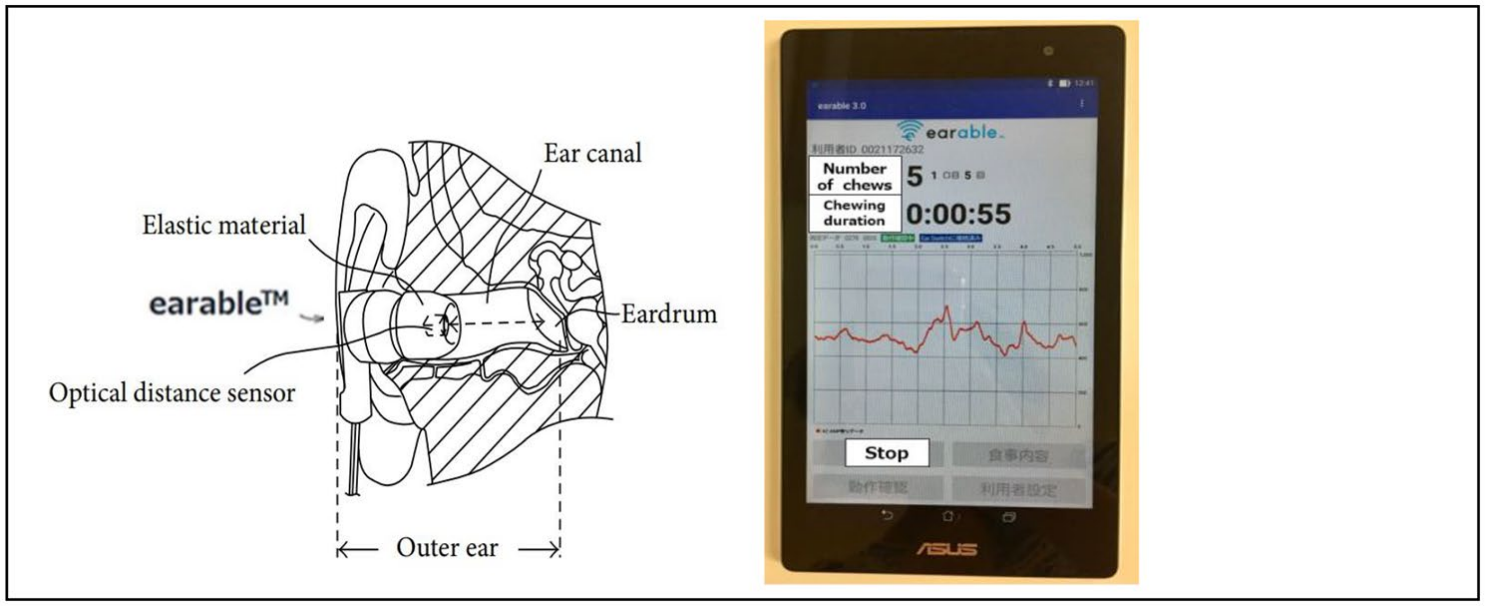
Structure of the “earable” device and tablet screen

Mastication was evaluated by attaching an earphone-type masticatory meter called “earable” (eRCC, Hiroshima, Japan) to the outer ear opening. It is plugged into the opening of the external auditory canal and utilizes infrared light. In addition, an optical distance sensor (LED and phototransistor) was installed to determine the number of chews and chewing duration by measuring the minute changes in the external auditory canal. The measured changes are displayed as waveforms; a proprietary algorithm is used to detect mastication and displays the mastication data on a tablet screen in real-time. Since the user is only required to wear an earphone, the device can measure masticatory indices under conditions closer to those experienced in everyday life than the conventional masticatory meters that are worn on the chin (Fig. 2).

This device has a precision of at least 0.958 and a recall of at least 0.937; therefore, it can accurately measure the number of chews [12].

For the evaluation of mastication, we used three food items [11]. The number of chews and chewing duration were measured during eating. The food items were characterized as follows:

(1) A salad (39 kcal, 1.2 g of protein, 0.5 g of fat, 6.2 g of carbohydrate, and 0.2 g of salt equivalent) contains ingredients, such as shredded cabbage, that are high in fiber. (2) Rice ball (188 kcal, 5.6 g of protein, 1.6 g of fat, 37.8 g of carbohydrate, and 0.9 g of salt equivalent) is a staple food in Japanese. (3) Donuts (214 kcal, 3.0 g of protein, 10.4 g of fat, 27.4 g of carbohydrate, 26.9 g of sugar, 0.5 g of dietary fiber, and 0.5 g of salt equivalent) are readily available snacks.

### Statistical analysis

The indices were expressed as the mean ± standard deviation or the median (1st–3rd quartiles). The Shapiro–Wilk test was used to test for normality. The pre-post-intervention comparison of the CTG and MIG was performed using the paired t-test or Wilcoxon signed-rank test. The pre-intervention values and changes in each indicator were compared using a two-sample t-test or Mann–Whitney U test. The analyses were performed using the Statistical Package for the Social Sciences version 25. A *p* value of < 0.05 was considered significant.

## Results

As regards the baseline patient background, the incidence of dyslipidemia was significantly higher in the MIG; however, no significant difference was observed in the other indices (Table 1).

**Table 1:** Participants’ characteristics

Measurements	Conventional treatment group (n = 12)	Mastication intervention group (n = 16)	*p* value
Age (years)	40.5 ± 12.7	44.6 ± 12.4	0.396
Height (cm)	158.5 ± 5.1	155.5 ± 6.2	0.187
Weight (kg)	93.6 ± 12.8	88.1 ± 20.5	0.419
Body fat percentage (%)	47.7 ± 4.7	47.7 ± 3.9	0.979
BMI (kg/m^2^)	37.4 ± 5.2	36.9 ± 6.7	0.854
Skeletal muscle mass (kg)	26.1 ± 3.2	25.4 ± 4.8	0.664
Body fat (kg)	45.6 ± 8.6	43.3 ± 12.54	0.590
Subcutaneous fat area (cm^2^)	497.0 ± 133.7	429.1 ± 159.3	0.243
Visceral fat area (cm^2^)	161.8 ± 67.6	175.6 ± 64.9	0.590
Diabetes n, (%)	1 (8.3)	6, (37.5)	0.184
Dyslipidemia n, (%)	0 (0.0)	6 (37.5)	0.024*
Hypertension n, (%)	2 (16.7)	3 (18.8)	1.000
Fatty liver disease n, (%)	1 (8.3)	5 (31.5)	0.196
Smoking n, (%)	1 (8.3)	2 (12.5)	1.000
Drinking n, (%)	4 (33.3)	2 (12.5)	0.354
Exercise habits n, (%)	3 (25.0)	5 (31.5)	1.000

Data are presented as the mean ± standard deviation

*p < 0.05

BMI, body mass index

### Changes in masticatory indices

Among the masticatory and biochemical indices measured at baseline, the chewing duration was significantly different between the two groups only for salad (325.6 vs. 422.2, *p* = 0.035) (Table 2). The pre-post-intervention comparison revealed that both groups showed a significant increase in the chewing duration and number of chews when eating salad (Table 2). The MIG showed a significant increase in the chewing duration (from 242.7 ± 90.8 to 273.6 ± 79.1 s, *p* = 0.040) and number of chews (from 205.0 [166.0–336.3] to 298.0 [221.0–379.3] times, *p* = 0.006) for rice ball and a significant increase in the number of chews for donuts (from 149.5 [119.8–215.5] to 210.0 [155.5–255.0] times, *p* = 0.011). However, these significant changes were not observed in the CTG. No significant difference was observed in the rate of change of each index between the two groups.

**Table 2:** Changes in masticatory indices according to the food item consumed before and after 6 months of intervention

	Conventional treatment group			Mastication intervention group		
**Measurements**	**Baseline** **(n = 12)**	**After 6 months** **(n = 12)**	***p *** *value*	**Baseline** **(n = 16)**	**After 6 months** **(n = 16)**	***p *** *value*
Chewing duration for salad (s)	325.6 (262.8–344.0)	402.0(269.3–493.3)	0.041*^,†^	422.2 ± 140.4^§^	499.8 ± 188.4	0.020*
Number of chews for salad (times)	326.3 ± 109.9	466.4 ± 204.9	0.056	432.3 ± 165.5	544.8 ± 243.5	0.023*
Chewing duration for rice ball (s)	230.8 ± 75.4	247.3 ± 79.8	0.289	242.7 ± 90.8	273.6 ± 79.1	0.040*
Number of chews for rice ball (times)	246.1 ± 99.3	283.7 ± 98.6	0.327	205.0 (166.0–336.3)	298.0(221.0-379.3)	0.006**^,†^
Chewing duration for a donut (s)	167.5(145.3–191.0)	171.5(149.8–251.0)	0.367^†^	186.8 ± 49.3	210.3 ± 67.5	0.114
Number of chews for a donut (times)	189.5 ± 82.7	202.8 ± 50.4	0.613	149.5 (119.8–215.5)	210.0(155.5–255.0)	0.011*^,†^

^†^Wilcoxon signed-rank test was performed

Data are presented as the mean ± standard deviation or the median (in the 25–75% range)

*p < 0.05, **p < 0.01

§*p* < 0.05 vs. before the conventional treatment group

### Changes in body composition and biochemical indicators

The changes in body composition and biochemical indices after the 6-month intervention provided in both groups are presented in Table 3. Both groups showed significant decreases in body weight, BMI, skeletal muscle mass, body fat, and visceral fat area. Furthermore, the subcutaneous fat area (from 497.0 ± 133.7 to 408.8 ± 166.4 cm^2^, *p* = 0.015) in the CTG and body fat percentage (from 47.7 ± 3.9 to 45.0 ± 4.8%, *p* < 0.001) in the MIG significantly decreased. Among the biochemical indices, the HbA1c, AST, ALT, and GGT levels in both groups significantly decreased, while the LDL and HDL levels significantly increased.

**Table 3:** Changes in body composition and biochemical indices before and after 6 months of intervention

	Conventional treatment group			Mastication intervention group		
**Measurements**	**Baseline** **(n = 12)**	**After 6 months** **(n = 12)**	***p *** *value*	**Baseline** **(n = 16)**	**After 6 months** **(n = 16)**	***p *** *value*
Weight (kg)	93.6 ± 12.8	89.8 ± 13.9	0.005*	88.1 ± 20.5	82.3 ± 18.1	0.030*
Body fat percentage (%)	47.7 ± 4.7	47.2 ± 5.2	0.729	47.7 ± 3.9	45.0 ± 4.8	< 0.001**
BMI (kg/m^2^)	37.4 ± 5.2	35.8 ± 5.5	0.003*	36.9 ± 6.7	33.8 ± 6.1	< 0.001**
Skeletal muscle mass (kg)	26.1 ± 3.2	25.6 ± 3.1	0.005*	25.4 ± 4.8	24.3 ± 4.5	< 0.001**
Body fat (kg)	45.6 ± 8.6	42.6 ± 10.3	0.016*	43.3 ± 12.5	37.6 ± 11.6	< 0.001**
Subcutaneous fat area (cm^2^)	497.0 ± 133.7	408.8 ± 166.4	0.015*	429.1 ± 159.3	391.5 ± 157.2	0.154
Visceral fat area (cm^2^)	161.8 ± 67.6	135.1 ± 93.2	0.041*	175.6 ± 64.9	150.7 ± 60.7	0.019*
HbA1c (%)	5.8 (5.5–6.1)	5.8 (5.4–5.9)	0.027*^,†^	6.0 ± 0.6	5.8 ± 0.5	0.014*
AST (IU/L)	21.50 (17.8–31.3)	18.0 (16.0–24.5)	0.020*^,†^	30.0 (17.0–35.8)	18.0 (17.0–24.8)	0.022*^,†^
ALT (IU/L)	28.0 (17.8–39.3)	16.5 (14.0–29.8)	0.008*^,†^	36.5 (20.3–55.3)	22.0 (15.8–36.3)	0.008**^,†^
GGT (IU/L)	32.5 ± 15.2	24.6 ± 10.1	0.005**	37.6 ± 17.2	25.1 ± 12.9	0.002**
FPG (mg/dL)	104.5(91.3–109.0)	99.5 (95.8–106.5)	0.929^†^	107.4 ± 17.2	98.1 ± 12.5	0.007**
TG (mg/dL)	110.5(82.5–162.3)	91.5 (73.5–131.0)	0.002*^,†^	116.5(76.3-224.8)	87.0 (61.0–129.3)	0.059^†^
HDL (mg/dL)	45.4 ± 9.5	57.4 ± 15.1	< 0.001**	41.0 (35.0–49.0)	48.5 (43.3–56.5)	0.001**^,†^
LDL (mg/dL)	113.0 ± 28.3	126.0 ± 20.9	0.049*	111.1 ± 33.1	119.8 ± 31.3	0.013*
IRI (µU/mL)	11.0 (7.1–21.3)	14.9 (10.0–24.7)	0.272^†^	12.5 (9.1–18.2)	9.9 (6.2–16.9)	0.215^†^
HOMA-R	3.8 ± 2.3	4.2 ± 1.9	0.551	3.7 (2.1–3.9)	2.2 (1.5–4.0)	0.109^†^

^†^Wilcoxon signed-rank test was performed

*p < 0.05, **p < 0.01

Data are presented as the mean ± standard deviation or the median (in the 25–75% range)

AST, aspartate aminotransferase; ALT, alanine aminotransferase; GGT, gamma-glutamyl transferase; FPG, fasting plasma glucose; HbA1c, hemoglobin A1c; TG, triglyceride; LDL, low-density lipoprotein; HDL, high-density lipoprotein; IRI, immunoreactive insulin; HOMA-R, homeostasis model assessment of insulin resistance

**Fig. 3:**
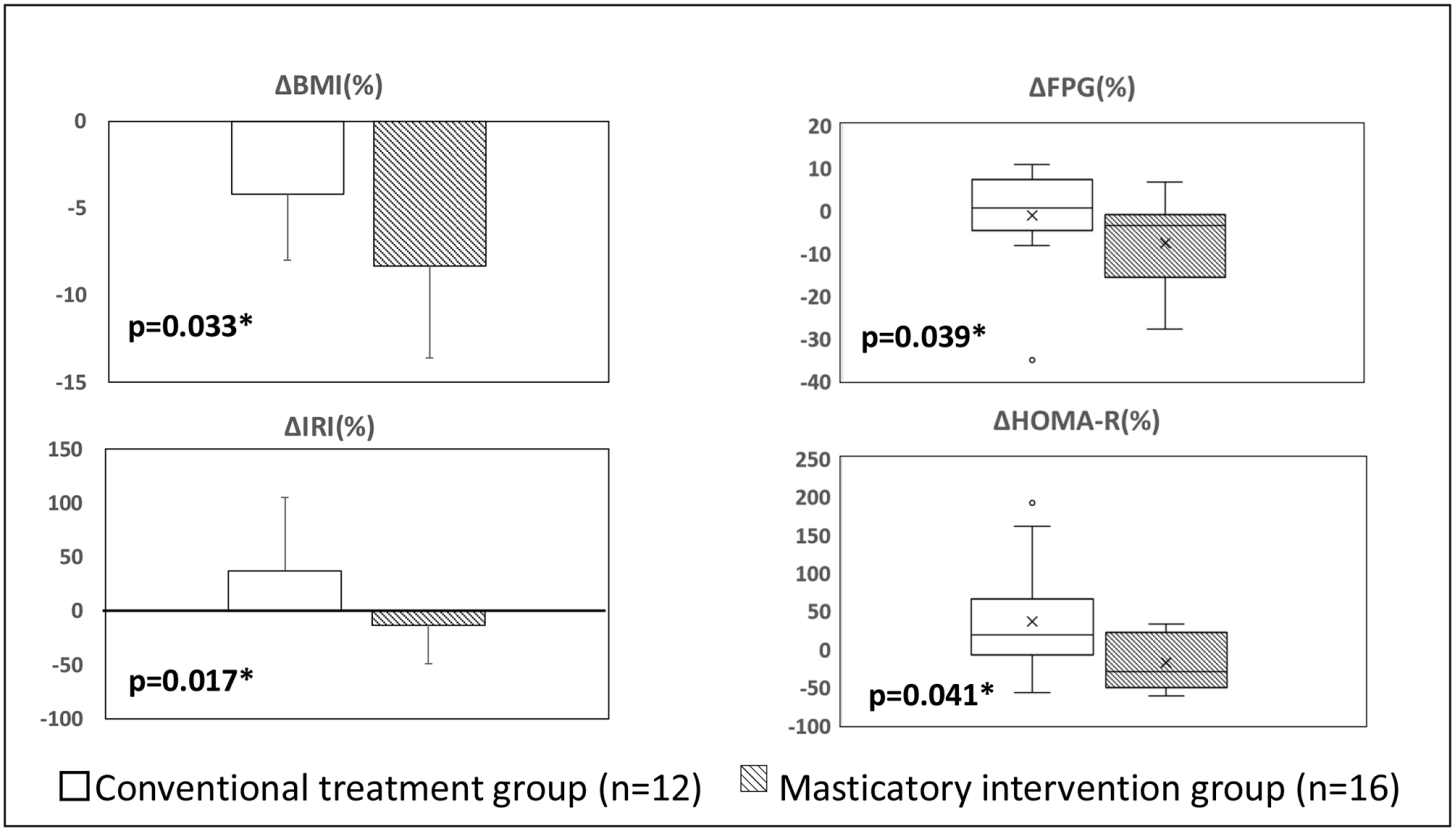
Comparison of the rate of changes in body composition and biochemical indicators between the CTG and MIG

Furthermore, the levels of TG (from 110.5 [82.5–162.3] to 91.5 [73.5–131.0] mg/dL, *p* = 0.002) in the CTG and FPG (from 107.4 ± 17.2 to 98.1 ± 12.5 mg/dL, *p* = 0.007) in the MIG significantly decreased. When the rates of change in body composition and biochemical indices were compared, the BMI (− 4.2 ± 3.8 vs. −8.3 ± 5.3%, *p* = 0.033), FPG (− 0.5 [− 4.8 to 7.1] vs. −3.6 [− 15.8 to − 1.1]%, *p* = 0.039), IRI (37.3 ± 68.2 vs. −13.4 ± 35.4%, *p* = 0.017), and HOMA-R values (− 18.4 [− 7.9 to 65.8] vs. −31.5 [− 49.1 to 21.3]%, *p* = 0.041) significantly improved in the MIG compared with that in the CTG (Fig. 3).

## Discussion

The masticatory indices for salad improved in both groups. By contrast, the number of chews and chewing duration for rice ball and number of chews for donuts also improved in the MIG. With regard to the body composition indices, the body weight, BMI, skeletal muscle mass, and body fat mass improved, and the HDL and LDL levels increased in both groups after 6 months of intervention. Meanwhile, the body fat percentage and FPG significantly decreased in the MIG. Among the biochemical indices, the HbA1c, AST, ALT, and GGT values improved significantly in both groups. Based on the rates of change between the two groups, the BMI, FPG, IRI, and HOMA-R values in the MIG were significantly lower than those in the CTG. In both groups, the content of nutritional and exercise guidance provided was the same; this result is considered an additional effect when masticatory guidance was added.

A previous study reported that chewing gum between meals alone did not promote weight loss [13]. Quantification of the number of chews and duration of chewing foods that are frequently consumed in daily life and provision of guidance on mastication are important.　Another previous study reported that an increase in the number of times a person chews a fixed-amount meal reduces short-term appetite, and regulates glucose metabolism [14]. Our findings showed that masticatory guidance increased the number of chews and chewing duration during the intake of rice ball and donuts, leading to a decrease in the body fat, BMI, and FPG by improving the manner in which staple foods are consumed in daily life. Suzuki et al. reported that the adequate chewing of test foods suppressed the postprandial increase in blood glucose levels and improved the early-phase insulin response [15]. The same mechanism was considered in this study, the increase in the number of chews and duration of chewing carbohydrates suppressed the rapid elevation of blood glucose levels and contributed to the improvement of glucose metabolism. Another possible mechanism is the effect of histamine secretion due to mastication. An increase in the number of chews promotes histamine secretion and prevents overeating by stimulating the satiety center [16, 17]. Fujise et al. reported that mastication activated the metabolism of histamine [18]. The increase in the number of chews and duration of chewing may have corrected overeating via the secretion of histamine in the brain and stimulation of the satiety center, leading to the reduction in body fat and BMI. Moreover, the increase in the number of chews may have affected the secretion of gastrointestinal hormones by decreasing the ghrelin levels and increasing the glucagon-like peptide-1 levels [10], promoting insulin secretion, and reducing the energy intake by inhibiting food intake and inducing a sense of satiety. However, increase in the number of chews may also have contributed to weight loss and improvement of glucose metabolism.

In addition to regular nutritional guidance, the mastication intervention encouraged patients to recognize their masticatory behavior by measuring their masticatory indices in advance. Furthermore, the patients were encouraged to change their behavior by providing individual feedback and guidance on mastication. It is conceivable that because of this intervention, although chewing instruction was provided once a month by a registered dietician, changes in chewing behavior were observed after 6 months. This was expected to influence the three daily meals. Specifically, this study quantified the number of chews and chewing duration during nutritional guidance and provided valuable results regarding the usefulness of chewing guidance along with the results. The Japanese Ministry of Health, Labor and Welfare recommends chewing each mouthful of food 30 times, which has been implemented in many studies [19].

After giving feedback on the number and duration of mastication before intervention, we instructed the participants to increase the number of mastications. Since the number and duration of mastications vary depending on the food item consumed, it is necessary to provide guidance on how to select food, how to cook it, and how to reduce the size of each bite. The recommended number and duration of mastications for each ingredient have not been reported in this study and should be investigated in future studies.

This study has some limitations. First, only female patients with obesity were included in this study. Therefore, whether the same results would be observed in male patients with obesity remains unknown. Previous studies have reported sex-related differences in mastication characteristics [11, 20]. A study comparing the effect of mastication index in men and women is needed. Second, this study was conducted by calculating the sample size; however, three dropouts occurred, and the verification power was relatively small. Moreover, the pre-post-intervention changes in total energy intake are unknown. The total energy intake was estimated based on the information provided by the participants during interviews with dietitians, although it was unclear. Therefore, the quantification of energy intake using questionnaires and application-based calculations is needed in future studies. Third, the participants’ intake of different nutrients was also unknown. The balance of intakes of carbohydrate, fat, protein, mineral, and vitamin also strongly influences weight loss. This study showed increased masticatory indices for staple foods, which may be observed among patients with a high carbohydrate intake. The MIG chewing index significantly improved after the intervention, but no significant difference was found in the rate of change between the CTG and MIG. This may be due to the large variability in the number of chewing times before intervention for rice balls and donuts. As the difference from the CTG was not clear, the group probably received general nutritional guidance for weight loss, and habituation possibly influenced the results of the second evaluation. In the end, since the actual meals are taken three times a day, behavioral changes owing to mastication instruction were believed to have been cumulative and contributed to the effects on body composition and glucose metabolism.

## Conclusions

We quantified the number of chewing times and chewing duration for obese women, and found that providing chewing guidance significantly improved the BMI, glucose metabolism, and insulin resistance than the regular nutritional guidance. In particular, the chewing instructions for carbohydrates such as rice balls and donuts may contribute to the improvement of sugar metabolism. Notably, increasing the number of chews and chewing duration while consuming carbohydrates, such as rice balls and donuts, may contribute to the improvement of glucose metabolism.

## Data Availability

The datasets generated and/or analyzed during the study are not publicly available due to ethical concerns but are available from the corresponding author upon reasonable request.

## List of Abbreviations

BMI: Body mass index
CTG: Conventional treatment group
MIG: Mastication intervention group
CT: Computed tomography
AST: Aspartate aminotransferase
ALT: Alanine aminotransferase
GGT: Gamma-glutamyl transferase
FPG: Fasting plasma glucose
HbA1c: Hemoglobin A1c
TG: Triglyceride
LDL: Low-density lipoprotein
HDL: High-density lipoprotein
IRI: Immunoreactive insulin
HOMA-R: Homeostasis model assessment of insulin resistance.
